# Supplementary material for: Impact of the COVID-19 pandemic on routine HIV care and antiretroviral treatment outcomes in Kenya: A nationally representative analysis
Source: PLoS One. 2023 Nov 27;18(11):e0291479. doi: 10.1371/journal.pone.0291479 (PMC10681195; doi:10.1371/journal.pone.0291479)
Supplement: S1 Table — (PDF) [file pone.0291479.s001.pdf]

SUPPLEMENTARY TABLES

S1 Table.

**Characteristics**		**Apr 2018 To Mar 2019 (n=2,505)**	**Apr 2019 To Mar 2020 (n=2,538)**	**Apr 2020 To Mar 2021 (n=2,003)**	**Overall (n=7,046)**
**Gender**	Female	1,681 (67.1)	1,692 (66.7)	1,330 (66.4)	4,703 (66.7)
	Male	824 (32.9)	846 (33.3)	673 (33.6)	2,343 (33.3)
**Age group (years)**	15.0 – 24.9	430 (17.2)	434 (17.1)	344 (17.2)	1,208 (17.1)
	25.0 – 34.9	909 (36.3)	931 (36.7)	718 (35.9)	2,558 (36.3)
	35.0 – 44.9	652 (26.0)	666 (26.2)	577 (28.8)	1,895 (26.9)
	45.0 – 54.9	337 (13.5)	343 (13.5)	249 (12.4)	929 (13.2)
	55.0+	177 (7.1)	164 (6.5)	115 (5.7)	456 (6.5)
**First-line ART regimen**	NVP-based	51 (2.0)	3 (0.1)	1 (0.1)	55 (0.8)
	EFV-based	1,375 (54.9)	863 (34.0)	121 (6.0)	2,359 (33.5)
	DTG-based	365 (14.6)	1,088 (42.9)	1,658 (82.8)	3,111 (44.2)
	Others	34 (1.4)	20 (0.8)	7 (0.4)	61 (0.9)
	Missing	680 (27.2)	564 (22.2)	216 (10.8	1,460 (20.7)
**HIV diagnosis to ART initiation (days)**	Same day	1,235 (49.3)	1,308 (51.5)	1,294 (64.6)	3,837 (54.5)
	1-14 days	212 (8.5)	189 (7.5)	149 (7.4)	550 (7.8)
	15-90 days	165 (6.6)	128 (5.0)	86 (4.3)	379 (5.4)
	91+ days	333 (13.3)	197 (7.8)	97 (4.8)	627 (8.9)
	Missing	560 (22.4)	716 (28.2)	377 (18.8)	1,653 (23.5)
**ART start to initial viral load (months)**	<3.0	121 (4.8)	100 (3.9)	71 (3.5)	292 (4.1)
	3.0 – 5.9	459 (18.3)	464 (18.3)	253 (12.6)	1,176 (16.7)
	6.0 – 8.9	589 (23.5)	635 (25.0)	240 (12.0)	1,464 (20.8)
	9.0 – 11.9	144 (5.8)	187 (7.4)	33 (1.7)	364 (5.2)
	<Missing	1,192 (47.6)	1,152 (43.4)	1,406 (70.2)	3,750 (53.2)
