# Supplementary material for: Impact of the COVID-19 pandemic on routine HIV care and antiretroviral treatment outcomes in Kenya: A nationally representative analysis
Source: PLoS One. 2023 Nov 27;18(11):e0291479. doi: 10.1371/journal.pone.0291479 (PMC10681195; doi:10.1371/journal.pone.0291479)
Supplement: S2 Table — (PDF) [file pone.0291479.s002.pdf]

S2 Table.

Characteristics		Same day, n (%)	Crude RR (95% CI)	p-value	Adjusted RR (95% CI)	p-value
**Pandemic periods**	Pre-COVID-19	2,543 (67.5)	Ref		Ref	
	COVID-19	1,294 (79.6)	1.16 (1.12 – 1.20)	<0.001	1.09 (1.04 – 1.13)	<0.001
**Gender**	Female	2,599 (71.8)	Ref		Ref	
	Male	1,238 (69.8)	0.97 (0.93 – 1.00)	0.065	0.97 (0.93 – 1.00)	0.065
**Age group (years)**	15.0 – 24.9	759 (78.8)	1.18 (1.09 – 1.28)		1.15 (1.07 – 1.25)	
	25.0 – 34.9	1,435 (72.3)	1.10 (1.02 – 1.19)		1.08 (1.01 – 1.17)	
	35.0 – 44.9	985 (69.1)	1.06 (0.98 – 1.14)		1.04 (0.97 – 1.13)	
	45.0 – 54.9	437 (64.1)	1.00 (0.91 – 1.09)		0.98 (0.90 – 1.07)	
	55.0+	221 (65.4)	Ref	<0.001	Ref	<0.001
**First-line ART regimen**	EFV-based	1,184 (69.4)	Ref		Ref	
	DTG-based	1,865 (76.7)	1.09 (1.05 – 1.13)		1.07 (1.02 – 1.13)	
	Others	6 (11.3)	0.18 (0.07 – 0.47)		0.18 (0.07 – 0.49)	
	Missing	782 (65.0)	0.92 (0.88 – 0.96)	<0.001	0.93 (0.89 – 0.98)	<0.001

*Missing date of HIV diagnosis (n=1,653 [23.5%]).
