# Supplementary material for: Impact of the COVID-19 pandemic on routine HIV care and antiretroviral treatment outcomes in Kenya: A nationally representative analysis
Source: PLoS One. 2023 Nov 27;18(11):e0291479. doi: 10.1371/journal.pone.0291479 (PMC10681195; doi:10.1371/journal.pone.0291479)
Supplement: S3 Table — (PDF) [file pone.0291479.s003.pdf]

S3 Table.

Characteristics		Attrition, d/Y (rate per 100 pyo)	Crude HR (95% CI)	p-value	Adjusted HR (95% CI)	p-value
**Pandemic periods**	Pre-COVID-19	1,448/149.5 (9.69)	Ref		Ref	
	COVID-19	394/90.8 (4.34)	0.54 (0.49 – 0.61)	<0.001	0.66 (0.58 – 0.74)	<0.001
**Gender**	Female	1190/161.9 (7.35)	Ref		Ref	
	Male	652/78.5 (8.31)	1.12 (1.02 – 1.23)	0.019	1.19 (1.07 – 1.32)	0.001
**Age group (years)**	15.0 – 24.9	334/38.9 (8.58)	1.08 (0.88 – 1.33)			
	25.0 – 34.9	681/88.6 (7.69)	1.01 (0.83 – 1.22)			
	35.0 – 44.9	482/64.5 (7.48)	0.96 (0.79 – 1.18)			
	45.0 – 54.9	220/32.5 (6.77)	0.86 (0.69 – 1.07)			
	55.0+	125/15.9 (7.87)	Ref	0.115	-	
**First-line ART regimen**	EFV-based	600/79.5 (7.55)	Ref		Ref	
	DTG-based	610/115.3 (5.29)	0.72 (0.64 – 0.81)		0.82 (0.72 – 0.94)	
	Others	34/3.5 (9.75)	1.13 (0.80 – 1.59)		1.16 (0.82 – 1.64)	
	Missing	598/42.0 (14.23)	1.91 (1.70 – 2.14)	<0.001	1.98 (1.76 – 2.23)	<0.001
**Same day HIV diagnosis and ART start**	No	360/55.6 (6.47)	Ref		Ref	
	Yes	1000/133.3 (7.50)	1.16 (1.03 – 1.31)		1.35 (1.19 – 1.52)	
	Missing	482/51.4 (9.38)	1.33 (1.15 – 1.53)	<0.001	1.50 (1.30 – 1.73)	<0.001
