# Supplementary material for: Impact of the COVID-19 pandemic on routine HIV care and antiretroviral treatment outcomes in Kenya: A nationally representative analysis
Source: PLoS One. 2023 Nov 27;18(11):e0291479. doi: 10.1371/journal.pone.0291479 (PMC10681195; doi:10.1371/journal.pone.0291479)
Supplement: S4 Table — (PDF) [file pone.0291479.s004.pdf]

S4 Table.

Characteristics		VnS, n (%)	Crude OR (95% CI)	p-value	Adjusted OR (95% CI)	p-value
**Pandemic periods**	Pre-COVID-19	225/2,699 (8.3)	Ref		Ref	
	COVID-19	32/597 (5.4)	0.62 (0.43 – 0.91)	0.015	0.79 (0.52 – 1.20)	0.264
**Gender**	Female	182/2,241 (8.1)	Ref			
	Male	75/1,055 (7.1)	0.87 (0.65 – 1.14)	0.312	-	-
**Age group (years)**	15.0 – 24.9	50 (10.1)	2.96 (1.38 – 6.35)		2.54 (1.18 – 5.20)	
	25.0 – 34.9	101 (8.3)	2.37 (1.14 – 4.94)		2.10 (1.00 – 4.41)	
	35.0 – 44.9	67 (7.4)	2.11 (1.00 – 4.46)		1.97 (0.93 – 4.19)	
	45.0 – 54.9	31 (6.7)	1.87 (0.85 – 4.14)		1.87 (0.84 – 4.15)	
	55.0+	8 (3.7)	Ref	0.042	Ref	0.134
**First-line ART regimen**	EFV-based	118/1,190 (9.9)	Ref		Ref	
	DTG-based	71/1,348 (5.3)	0.51 (0.37 – 0.69)		0.58 (0.42 – 0.82)	
	Others	6/56 (10.7)	1.09 (0.46 – 2.60)		1.16 (0.48 – 2.77)	
	Missing	62/702 (8.8)	0.88 (0.64 – 1.22)	<0.001	0.93 (0.67 – 1.29)	0.010
**Same day HIV diagnosis and ART start**	No	68/828 (8.2)	Ref			
	Yes	124/1,772 (7.0)	0.84 (0.62 – 1.14)			
	Missing	65/696 (9.3)	1.15 (0.81 – 1.64)	0.132	-	-
**Duration from ART start to initial viral load test (months)**	<3.0	24/292 (8.2)	Ref			
	3.0 – 5.9	93/1,176 (7.9)	0.96 (0.60 – 1.53)			
	6.0 – 8.9	116/1,464 (7.9)	0.96 (0.61 – 1.52)			
	9.0 – 11.9	24/364 (6.6)	0.79 (0.44 – 1.42)	0.836	-	-

*HIV viral load test not yet done (n=3,750 [53.2%])
